# Behavioral and Psychosocial Dynamics of Engagement: The Digital Divide in Artificial Intelligence [AI]-Driven Sports Podcasts

**DOI:** 10.3390/bs14100911

**Published:** 2024-10-08

**Authors:** Yair Galily, Tal Laor, Tal Samuel Azran

**Affiliations:** 1 School of Communications, Reichman University, Herzliya 4610101, Israel; tazran@runi.ac.il; 2 School of Communications, Ariel University, Ariel 4070000, Israel; tall@ariel.ac.il

**Keywords:** sport, AI, podcasts

## Abstract

The digital divide, particularly within the context of Artificial Intelligence (AI) sport podcasts, presents significant behavioral and psychosocial challenges for student engagement. This study examines the disparities in access to and proficiency with Information Communication Technologies (ICTs) across different demographic groups, focusing on gender, age, and religious level. The advent of the commercial web has heightened the significance of these divides, as the first-level digital divide concerns access to the internet, while the second-level digital divide pertains to the ability to use technology proficiently. The existing literature has consistently highlighted persistent inequalities in these areas, which significantly impact the extent to which students from various backgrounds can engage with AI sport podcasts effectively. Understanding these dynamics is crucial for developing strategies to bridge the gap and ensure equitable access to digital learning resources.

## 1. Introduction

The ability of different demographics to gain equal access and benefits from Information communication technologies (ICTs) has occupied scholars for decades [1,2]. With the advent of the commercial web, analyses have focused on access to the internet, namely the first-level digital divide [3], and the ability to use the technology in a skilled manner, namely the second-hand digital divide [4]. Three of the main demographics usually analyzed for the divide include gender [5,6], age [7], and religious level [8], with the majority of studies identifying persistent divides for all three groups [9].

Whereas studies so far examined the issue with respect to the general population, not enough studies examined the digital divide theory with respect to students and none have examined it with respect to the usage of Artificial Intelligence. This study aims to contribute an innovative analysis to the current literature by examining which student demographics, specifically with respect to gender, age, and religious level, listen to AI sport podcasts. (Artificial Intelligence (AI) is rapidly transforming various industries, and the world of sports is no exception. From football to cricket to Formula 1, Al is reshaping how athletes train, how teams strategize, and how fans experience the game. The impact of AI on sports is multi-faceted, reaching far beyond the pitch or track.) Gaps in the usage of AI at the university level are critical as a McKinsey report predicts that by 2030, 400 million people could be displaced because of AI platforms who will fill their position [10] while the World Economic Forum predicts that AI will contribute to create 97 million new jobs [11], thus making familiarity with AI platforms crucial for success in the academia and in the workforce.

Indeed, there are already early signs for emergent gaps, specifically the gender gap in academia on the level of university faculty. The World Economic Forum identified that only 13.83% of Artificial Intelligence-related papers are authored by women and only 18% of the authors at the main AI conferences are women [12]. This trend is mirrored in the industry level as well, as only 2% of venture-capital-funded women entrepreneurs start AI ventures and women form only 22% of the AI global workforce. In contrast to the gender gap within the faculty, analysis within the student body might reflect other trends, such as adoption trends, which might be different than the traditional digital divide patterns.

To conduct our study, we surveyed students at a private Israeli university about their exposure to AI sport content, specifically through podcasts, and their purposes for engaging with this content. People listen to AI-driven sports podcasts for convenience, in-depth analysis, and personalized content. These podcasts provide real-time updates, expert insights, and tailored discussions, allowing fans to stay connected to their favorite teams and sports while on the go. The AI-driven element enhances the experience by offering customized recommendations and insights, making it easier for listeners to engage with niche topics or stay informed on trends that matter to them [13].

We received responses from 207 students, which university staff then analyzed with particular emphasis on gender, religious level, and age. Previous studies have successfully used podcast listening patterns to evaluate the digital divide concerning technology [14].

### 1.1. Gender Digital Divide and AI

The choice of AI-related content as a measure of gender disparity in academia stems from its crucial role for any assessment of students’ future career in light of its apparent role in leading a fourth industrial revolution [14]. The question of gender equality in the science and technology fields played a central role in studies for decades [15,16]. Mostly, studies highlight a disproportionate advantage for men in all science, Technology, Engineering, and Math (STEM) realms in terms of career choice, rank, and skills [17,18]. In academia, where students usually begin their career paths, a recent study found that the percentage of males graduating informational and communication technologies programs is four times higher than females [19]. Importantly, studies that explain this gender gap are based on analyses that reveal that women succumb to their gender stereotype about their inherent lower abilities in the STEM fields from the age of six [20,21,22]. This happens even when in reality their performance in exams shows that their abilities are evidently high in these fields [23]. Instead, they often choose non-STEM courses such as Nursing, Education, and Communication studies [24,25], courses in which the majority of students are traditionally female and where wages are relatively lower than STEM.

In contrast, some studies in the developed world found no significant gender differences with respect to STEM access and skills. In Switzerland, a study of high school students’ technological skills found minimal gender differences [26]. In a study conducted in Canada, there were no differences between the male and female students in the department in terms of skills [27]. Similarly, in Spain, a study of the gendered differences in technical and computer skills of teachers revealed no significant differences between female and male teachers [28].

### 1.2. Age and the Digital Divide

Overall, there is a near consensus that age plays a major role in access and use of technology as well as in difference in skills [29,30,31]. Studies have found that young adults were less anxious about technology than middle-aged adults and that older adults were more anxious about technology than middle-aged adults, drawing a clear link between age and technology use [32]. A major study in Switzerland further highlighted the so-called “grey divide” between people over 65 years of age and indicated that older seniors over 70 years of age are often excluded from web usage and learning new skills [33]. The problem of technophobia indeed appears to grow in a linear way with age as studies conducted on the interplay between age and the use of the web indicate that with every 5 years of age, particularly above the age of 65, the likelihood of using the web decreases by 8% [34]. Other studies [34] also distinguish between digital immigrants and digital natives, with the first being at a major disadvantage as opposed to the younger generations growing up with computers and the web [35].

A minority study conducted in Canada and Australia argued that studies of age and the digital divide ignore the social context of the individuals and treats them as a homogenous group. The study argues that gender, practices, social class, social norms, and other factors should be taken into account while analyzing the issue to better contextualize differences in this heterogenous group(s) [36].

### 1.3. Religiosity and the Digital Divide

Studies have identified that religiosity decreases the tendency to participate in online activities [37]. This is due both to the fact that the web presents a more secular prism of the world than many religious people would like to expose themselves to [38] as well as the fact that they tend to have stronger offline communities and are less in need of online communities [39]. Some studies identify that the effect is stronger for females [40].

In contrast, other studies argued that the web serves as a platform to reduce religious authority thanks to its anonymity, thus that religiosity will not have any effect on the tendency to use technology [41,42]. These studies actually see that the web serves as a perfect hub for religious fundamentalism, thus being used intensively by religious groups to promote their agendas [43].

## 2. Methodology

### 2.1. Procedure

The study was conducted following rigorous ethical guidelines, with approval secured from the Institutional Review Board (IRB) of the first author’s affiliated university in Israel. This approval was granted under the oversight of the University Research Ethics Committee, ensuring that all research activities met the necessary ethical standards for conducting studies involving human participants.

Data collection for this study was carried out through a structured survey, which was disseminated primarily via WhatsApp. The use of WhatsApp as a distribution platform allowed the researchers to reach a broad and diverse audience, including students at various stages of their academic careers—bachelor’s, master’s, and doctoral programs—as well as recent graduates. In addition to students, the survey was also distributed to administrative and academic staff members within a private Israeli university. This diverse sampling strategy was designed to capture a wide range of perspectives on the exposure to and engagement with content related to Artificial Intelligence (AI).

The central aim of the survey was to explore the extent to which these different groups are exposed to AI-related content, with a particular focus on podcasts as a medium. Moreover, the study sought to understand the purposes for which individuals engage with this content—whether for educational purposes, professional development, personal interest, or other reasons. The survey was carefully designed to gather detailed demographic information, allowing the researchers to analyze the data with a specific emphasis on variables such as gender, religious affiliation, and age. This demographic focus enabled a more nuanced understanding of how different groups within the university community interact with AI content.

The survey was conducted over a period in April 2024 and successfully obtained 203 completed responses. The sampling approach was one of convenience, meaning that the survey was available until the target number of responses was reached. Once this sample size was achieved, the survey was removed from the WhatsApp groups to conclude the data collection phase.

Participants were fully informed about the nature of the study and their role within it. It was explicitly communicated that participation was entirely voluntary and that all responses would remain anonymous. This anonymity was ensured to protect the privacy of the participants and to encourage honest and candid responses. Participants were also informed that they were not required to answer all questions and that they could choose to skip any question without any repercussions. Furthermore, the survey made it clear that no incentives, financial or otherwise, would be offered in exchange for participation. Before beginning the survey, participants were asked to acknowledge that they had read and understood these terms and agreed to proceed with the survey under these conditions.

The survey also included detailed information about the researchers conducting the study, including their names, affiliations, and contact details. This transparency was intended to provide participants with a clear understanding of who was responsible for the research and how they could reach out with any questions or concerns regarding the survey or the use of the data collected. This level of detail was provided to ensure that participants were fully informed and could make an educated decision about their participation in the study.

### 2.2. Measures

To explore the uses and gratifications associated with AI podcasts, the survey included questions addressing both socio-demographic characteristics and consumption patterns. The questionnaire was structured into two primary sections:

1. Demographics Section: Drawing on the principles of the digital divide and knowledge gap theory, this section comprised a series of closed-ended questions designed to assess a range of socio-demographic variables. These variables included gender, age, area of residence, religious affiliation, level of religiosity, marital status, number of children, level of education, and economic status, as outlined by [44] The intent was to provide a comprehensive demographic profile of the respondents, which could later be analyzed in relation to their podcast consumption habits.

2. Motives for AI Podcast Use: The second part of the questionnaire focused on understanding the motivations behind AI podcast consumption, utilizing [45] uses and gratifications framework. This section employed a two-step approach. Initially, participants were asked to rate the importance of various needs—categorized into affective, cognitive, and escapist needs—on a scale from 1 (not important at all) to 3 (very important). If a participant rated a need as even slightly important, they were then asked to evaluate how well AI podcasts helped satisfy that need, using a scale from 1 (not at all helpful) to 6 (very helpful).

Building on the uses and gratifications theory, this section included four key questions encompassing 29 statements that probed into the consumption habits and motivations of AI podcast listeners. For instance, participants were asked how frequently they listen to AI podcasts (daily, several times a week, several times a month, seldom, or not at all). They were also asked to identify the specific gratifications that AI podcasts provide, such as knowledge acquisition, escapism, coverage of topics not addressed by traditional media, added value, and social interaction. Additionally, the survey explored the contexts in which respondents consumed AI podcasts, such as while driving, during workouts, before bedtime, or while browsing the internet.

All questions were rated on a 6-point Likert scale, where 1 represented “not at all” and 6 represented “to a very large extent”. This scale was used to measure the extent of podcast consumption and the degree to which participants’ needs were met by listening to AI podcasts. Notably, the survey did not employ any subscales or psychometric indices, maintaining a straightforward approach to data collection.

To determine whether there were significant differences in the satisfaction of various needs based on socio-demographic factors (such as gender, age, and area of residence), the researchers conducted a one-way analysis of variance (ANOVA) followed by a Scheffé post-hoc analysis. This statistical approach allowed for the identification of any meaningful differences in podcast consumption patterns and motives across different demographic groups.

## 3. Findings

### 3.1. Demographics

There were 207 participants in the research; 65.2% of them were males and 34.8% were females. The average age was 35.35 years, with a median of 35 years and a standard deviation of 0.477. In terms of education level, most participants held a bachelor’s degree (43.5%), followed by those with a master’s degree or higher (33.3%). Regarding religious affiliation, the vast majority of participants were secular (75.4%). In terms of income level, most participants earned above the average income (69.6%), with the primary occupation being in the high-tech sector (25%).

Concerning podcast listening frequency, most participants listened between once a week and twice a week (42%), followed by those who listened between once a month and twice a month (31.9%).

### 3.2. Descriptive Statistics

Table 1 provides descriptive statistics related to listener needs for an AI podcast. The strongest needs were “provides added value” and “covers untraditional topics”, with means of 4.80 and 4.54, respectively; both medians were 5.0. Conversely, the lower scored needs were “provides escape” and “other needs” with means of 2.87 and 2.67, respectively.

Table 2 provides descriptive statistics related to the reasons for consuming the AI podcast. The strongest reasons were “the topics are relevant and up-to-date for me” and “the podcast is engaging and exciting”, with means of 5.04 and 4.42, respectively, and both having medians of 5.0. Conversely, the lowest scored reasons were “all my friends listen and recommend” and “topics are great for work discussions” with means of 2.17 and 3.41, respectively.

Table 3 provides descriptive statistics related to the various ways participants consume the AI podcast. The most common method was listening while driving, with a mean of 3.99 and a median of 5.00. Other frequent methods included listening while doing chores and during exercise, with means of 3.09 and 3.03, and medians of 3.00 and 2.00, respectively. The least common methods were listening before sleep (mean of 1.99, median of 1.00) and using it as background during work (mean of 2.01, median of 1.00).

Sex and Needs: (See Table 3)

1. To measure the relationship between the sex of the listener and the need for content that is not covered in traditional media, a Pearson test was conducted. The results indicated a weak correlation between the variables (r = 0.207, *p* < 0.01). This finding suggests that females tend to consume the podcast because of the need for content that is not covered in traditional media more than males, but this tendency is weak.
2. To measure the relationship between the sex of the listener and the other needs, a Pearson test was conducted. The results indicated a weak correlation between the variables (r = 0.212, *p* < 0.01). This finding suggests that females tend to consume the podcast because of other needs more than males, but this tendency is weak.
3. To measure the relationship between the sex of the listener and because the topics are “relevant and up-to-date” for the listener, a Pearson test was conducted. The results indicated a weak correlation between the variables (r = 0.162, *p* < 0.05). This finding suggests that females tend to consume the podcast because the topics are “relevant and up-to-date” for the listener more than males, but this tendency is weak.

Age (see Table 4):

1. To measure the relationship between age and the need for “knowledge that helps with my job”, a Pearson test was conducted. The test results indicated a weak positive correlation between the variables (r = 0.179, *p* < 0.01). This suggests that as age increases, so does the need to listen to the podcast for job-related information, but weakly.
2. To measure the relationship between age and the need for “knowledge that helps with personal life”, a Pearson test was conducted. The results showed a weak negative correlation between the variables (r = −0.262, *p* < 0.01). This suggests that as age increases, the need to listen to the podcast for achieving personal life-related information decreases, but weakly.
3. To measure the relationship between age and the need for consuming the podcast because it connects the person to a certain community, a Pearson test was conducted. The results showed a weak positive correlation between the variables (r = 0.210, *p* < 0.01). This suggests that as age increases, so does the need for content that connects to a certain community, but weakly.
4. To measure the relationship between age and the reason for listening to the AI podcast—”the podcast fascinates and excites me”—a Pearson test was conducted. The test results indicated a very weak positive correlation between the variables (r = 0.146, *p* < 0.01). This finding suggests that as age increases, the tendency to choose to listen to the podcast because it is fascinating and exciting also increases, but weakly.

Religiosity (see Table 5):

1. To measure the relationship between the religion status and the need for “knowledge that helps with personal life”, a Pearson test was conducted. The results showed a weak positive correlation between the variables (r = 0.256, *p* < 0.01). This suggests that as the level of religious affiliation (from secular to ultra-Orthodox) increases, so does the need to listen to the podcast for personal life-related information, but weakly.
2. To measure the relationship between the religion status and the response to the need for “escaping from the reality”, a Pearson test was conducted. The results showed a weak positive correlation between the variables (r = 0.162, *p* < 0.05). This suggests that as the level of religious affiliation (from secular to ultra-Orthodox) increases, so does the need to listen to the podcast to “escape from the reality”, but weakly.
3. To measure the relationship between the religion status and the need for content that is not covered in traditional media, a Pearson test was conducted. The results showed a weak positive correlation between the variables (r = 0.223, *p* < 0.01). This suggests that as the level of religious affiliation (from secular to ultra-Orthodox) increases, so does the need for content that is not covered in traditional media, but weakly.
4. To measure the relationship between the religion status and the need for consuming the podcast because it provides added value, a Pearson test was conducted. The results showed a weak positive correlation between the variables (r = 0.296, *p* < 0.01). This suggests that as the level of religious affiliation (from secular to ultra-Orthodox) increases, so does the need to listen to the podcast to gain added value, but weakly.
5. To measure the relationship between the religion status and the reason for listening to the podcast—“the podcast fascinates and excites me”, a Pearson test was conducted. The test results indicated a weak positive correlation between the variables (r = 0.207, *p* < 0.01). This suggests that as the level of religious affiliation (from secular to ultra-Orthodox) increases, so does the tendency to choose to listen to the podcast because it is fascinating and exciting, but weakly.
6. To measure the relationship between the religion status and listening to the podcast because of a connection to its presenter, a Pearson test was conducted. The test results indicated a weak positive correlation between the variables (r = 0.223, *p* < 0.01). This suggests that as the level of religious affiliation (from secular to ultra-Orthodox) increases, so does the tendency to choose to listen to the podcast because of a connection to its presenter, but weakly.
7. To measure the relationship between the religion status and listening to the podcast because the topics are “relevant and up-to-date” for the listener, a Pearson test was conducted. The test results indicated a weak negative correlation between the variables (r = 0.154, *p* < 0.05). This suggests that as the level of religious affiliation (from secular to ultra-Orthodox) increases, the tendency to choose to listen to the podcast because its content is relevant and up-to-date for the listener decreases, but weakly.

## 4. Discussion

The analysis explored the digital divide theory by examining the demographic characteristics of 207 Israeli students who listen to AI sports podcasts. The descriptive analysis revealed a notable gender disparity, with only about one-third of the listeners identifying as female. Similarly, the majority of respondents—approximately three-quarters—described themselves as secular. Interestingly, the average age of the participants was 45, suggesting that there is no significant digital divide when it comes to age in this context.

However, the results from the correlation analysis presented a more complex picture. Despite the overall weak correlations, the analysis revealed that participants from groups traditionally considered disadvantaged by digital divide theory—such as women, religious individuals, and older adults—reported using AI sports podcasts for personal and professional advancement more significantly than those from so-called advantaged groups. This finding, although subtle, suggests that members of these disadvantaged groups may be leveraging AI podcast content to gain an edge in areas where they might otherwise be at a disadvantage.

The overall findings present a mixed picture. Numerically, there appears to be a gender and religious/secular disparity in AI podcast listening patterns among students. This suggests that, in the realm of AI, the traditional gender gap and religious/secular divides persist, reflecting broader trends identified by digital divide theory. These findings are significant not only from a theoretical standpoint but also in terms of practical implications. They highlight the need for targeted programs aimed at closing these gaps. For instance, initiatives like “Girls Who Code” or “She Codes” could be adapted to focus on university students, particularly those from non-STEM disciplines, to enhance their coding and AI-related skills. Similarly, academic programs could be developed to increase access to AI education among religious students.

Regarding the weak correlation between religious, female, and older participants and their tendency to listen to AI podcasts for gaining an advantage, several explanations could be considered. One possibility is that individuals within these groups who do engage with AI podcasts may be “overachievers”, using these resources as a way to compensate for perceived disadvantages and striving to excel as a defense mechanism. Alternatively, the weak correlations might reflect a broader methodological issue: the failure to adequately account for the diverse social contexts of the individuals within these groups, rather than treating them as homogenous categories. This perspective is supported by the work of Neves, who emphasizes the importance of understanding the nuanced social contexts of individuals when analyzing such data.

## 5. Conclusions

The study examined the digital divide theory by analyzing the demographic characteristics of 207 Israeli students who listen to AI-generated sports podcasts. The descriptive findings highlighted a significant gender gap, with only about one-third of the listeners identifying as female. Additionally, the majority—around three-quarters—identified as secular. Interestingly, the average age of the participants was 35, indicating that age does not play a significant role in the digital divide in this context. Future research, following AI sport media research [46,47], should explore these hypotheses further, perhaps through qualitative methods like in-depth interviews. Such approaches could provide a more nuanced understanding of these unexpected findings, shedding light on the motivations and experiences of those who defy traditional patterns of digital divide theory in the context of AI podcast consumption.

## Figures and Tables

**Table 1:** Descriptive statistics—variable needs.

Statistics								
		Needs_Meets_Work_Related_Needs	Needs_Meets_Personal_Life_Needs	Needs_Provides_Escape	Needs_Covers_Untraditional_Topics	Needs_Provides_Added_Value	Needs_Connects_to_Community	Needs_Meets_Other_Needs
N	Valid	207	207	207	207	207	207	207
	Missing	0	0	0	0	0	0	0
Mean		3.7971	3.9710	2.8696	4.5362	4.7971	3.6667	2.6667
Median		4.0000	4.0000	2.0000	5.0000	5.0000	4.0000	2.0000
Std. Deviation		1.44390	1.50698	1.73132	1.49348	1.33925	1.63398	1.87472

**Table 2:** Descriptive statistics—variable reasons.

Statistics						
		Reasons_Is_Engaging	Reasons_Connection_to_Presenter	Reasons_Relevance	Reasons_Friends_Recommend	Reasons_Topics_Good_for_Work_Discussion
N	Valid	207	207	207	207	207
	Missing	0	0	0	0	0
Mean		4.4203	4.1449	5.0435	2.1739	3.4058
Median		5.0000	5.0000	5.0000	2.0000	3.0000
Std. Deviation		1.23547	1.59437	1.12476	1.36485	1.63088

**Table 3:** Descriptive statistics—ways of consumption.

		Way_of_Consumption_Exclusively	Way_of_Consumption_While_Driving	Way_of_Consumption_as_Work_Background	Way_of_Consumption_during_Exercise	Way_of_Consumption_While_Doing_Chores	Way_of_Consumption_before_Sleep	Way_of_Consumption_While_Browsing_pc	Way_of_Consumption_While_Browsing_Mobile
N	Valid	207	207	207	207	207	207	207	207
Mean		2.9420	3.9855	2.0145	3.0290	3.0870	1.9855	2.3188	2.1884
Median		3.0000	5.0000	1.0000	2.0000	3.0000	1.0000	1.0000	1.0000
Std. Deviation		1.64480	1.98655	1.45308	1.99736	1.78782	1.46307	1.70231	1.56672
Correlations									
							Gender_coded		
Needs_meets_work_related_needs					Pearson Correlation		0.039		
					Sig. (2-tailed)		0.572		
					N		207		
Needs_meets_personal_life_needs					Pearson Correlation		−0.067		
					Sig. (2-tailed)		0.338		
					N		207		
Needs_provides_escape					Pearson Correlation		−0.033		
					Sig. (2-tailed)		0.638		
					N		207		
Needs_covers_untraditional_topics					Pearson Correlation		0.207		
					Sig. (2-tailed)		0.003		
					N		207		
Needs_provides_added_value					Pearson Correlation		0.088		
					Sig. (2-tailed)		0.207		
					N		207		
Needs_connects_to_community					Pearson Correlation		0.075		
					Sig. (2-tailed)		0.285		
					N		207		
Needs_meets_other_needs					Pearson Correlation		0.212		
					Sig. (2-tailed)		0.002		
					N		207		
Reasons_is_engaging					Pearson Correlation		0.023		
					Sig. (2-tailed)		0.747		
					N		207		
Reasons_connection_to_presenter					Pearson Correlation		0.106		
					Sig. (2-tailed)		0.130		
					N		207		
Reasons_relevance					Pearson Correlation		0.162		
					Sig. (2-tailed)		0.020		
					N		207		
Reasons_friends_recommend					Pearson Correlation		−0.071		
					Sig. (2-tailed)		0.310		
					N		207		
Reasons_topics_good_for_work_discussion					Pearson Correlation		0.024		
					Sig. (2-tailed)		0.736		
					N		207		

Note: Correlation is significant at the 0.01 level (2-tailed). Correlation is significant at the 0.05 level (2-tailed).

**Table 4:** Age vs. needs.

Correlations		
		Age
Needs_meets_work_related_needs	Pearson Correlation	0.179
	Sig. (2-tailed)	0.010
	N	207
Needs_meets_personal_life_needs	Pearson Correlation	−0.262
	Sig. (2-tailed)	0.000
	N	207
Needs_provides_escape	Pearson Correlation	0.013
	Sig. (2-tailed)	0.858
	N	207
Needs_covers_untraditional_topics	Pearson Correlation	0.043
	Sig. (2-tailed)	0.540
	N	207
Needs_provides_added_value	Pearson Correlation	−0.031
	Sig. (2-tailed)	0.657
	N	207
Needs_connects_to_community	Pearson Correlation	0.210
	Sig. (2-tailed)	0.002
	N	207
Needs_meets_other_needs	Pearson Correlation	−0.071
	Sig. (2-tailed)	0.310
	N	207
Reasons_is_engaging	Pearson Correlation	0.146
	Sig. (2-tailed)	0.036
	N	207
Reasons_connection_to_presenter	Pearson Correlation	−0.073
	Sig. (2-tailed)	0.296
	N	207
Reasons_relevance	Pearson Correlation	0.033
	Sig. (2-tailed)	0.632
	N	207
Reasons_friends_recommend	Pearson Correlation	−0.056
	Sig. (2-tailed)	0.421
	N	207
Reasons_topics_good_for_work_discussion	Pearson Correlation	0.053
	Sig. (2-tailed)	0.452
	N	207

Note: Correlation is significant at the 0.01 level (2-tailed). Correlation is significant at the 0.05 level (2-tailed).

**Table 5:** Motivations to listen to sport podcasts.

Correlations		
		Religion
Needs_meets_work_related_needs	Pearson Correlation	−0.077
	Sig. (2-tailed)	0.268
	N	207
Needs_meets_personal_life_needs	Pearson Correlation	0.256
	Sig. (2-tailed)	0.000
	N	207
Needs_provides_escape	Pearson Correlation	0.162
	Sig. (2-tailed)	0.019
	N	207
Needs_covers_untraditional_topics	Pearson Correlation	0.223
	Sig. (2-tailed)	0.001
	N	207
Needs_provides_added_value	Pearson Correlation	0.296
	Sig. (2-tailed)	0.000
	N	207
Needs_connects_to_community	Pearson Correlation	0.104
	Sig. (2-tailed)	0.138
	N	207
Needs_meets_other_needs	Pearson Correlation	0.038
	Sig. (2-tailed)	0.585
	N	207
Reasons_is_engaging	Pearson Correlation	0.207
	Sig. (2-tailed)	0.003
	N	207
Reasons_connection_to_presenter	Pearson Correlation	0.223
	Sig. (2-tailed)	0.001
	N	207
Reasons_relevance	Pearson Correlation	0.154
	Sig. (2-tailed)	0.027
	N	207
Reasons_friends_recommend	Pearson Correlation	0.136
	Sig. (2-tailed)	0.051
	N	207
Reasons_topics_good_for_work_discussion	Pearson Correlation	0.065
	Sig. (2-tailed)	0.350
	N	207

Note: Correlation is significant at the 0.01 level (2-tailed). Correlation is significant at the 0.05 level (2-tailed).

## Data Availability

All statistical data is available upon request from the first author.

## References

[1] Elnaggar A. (2008). Towards gender equal access to ICT. Inf. Technol. Dev..

[2] Hoffman D.L., Novak T.P. (2000). The growing digital divide: Implications for an open research agenda. Underst. Digit. Econ. Data Tools Res..

[3] Ragnedda M., Kreitem H. (2018). The three levels of digital divide in East EU countries. World Media. J. Russ. Media Journal. Stud..

[4] Hargittai E. (2002). Second-level digital divide: Differences in People’s Online Skills. First Monday.

[5] Antonio A., Tuffley D. (2014). The gender digital divide in developing countries. Future Internet.

[6] Moshe M., Laor T., Fridkin S. (2017). Digital soap opera’online radio listening patterns and the digital divide. Isr. Aff..

[7] Abbey R., Hyde S. (2009). No country for older people? Age and the digital divide. J. Inf. Commun. Ethics Soc..

[8] Dilmaghani M. (2018). Religiosity and the digital divide in Canada. Commun. Rev..

[9] Abdelfattah B.M., Bagchi K., Udo G., Kirs P. Understanding the internet digital divide: An exploratory multi-nation individual-level analysis. Proceedings of the AMCIS 2010.

[10] Manyika J., Lund S., Chui M., Bughin J., Woetzel J., Batra P., Ko R., Sanghvi S. (2017). Jobs lost, jobs gained: Workforce transitions in a time of automation. McKinsey Glob. Inst..

[11] World Economic Forum How AI can help attract, nurture and retain talent. Proceedings of the World Economic Forum Annual Meeting.

[12] World Economic Forum (2022). Why We Must Act Now to Close the Gender Gap in AI. https://www.mckinsey.com/~/media/mckinsey/industries/public%20and%20social%20sector/our%20insights/what%20the%20future%20of%20work%20will%20mean%20for%20jobs%20skills%20and%20wages/mgi-jobs-lost-jobs-gained-executive-summary-december-6-2017.pdf.

[13] Samuel-Azran T., Laor T., Tal D. (2019). Who listens to podcasts, and why?: The Israeli case. Online Inf. Rev..

[14] Chakraborty U., Banerjee A., Saha J.K., Sarkar N., Chakraborty C. (2017). Artificial Intelligence and the Fourth Industrial Revolution.

[15] Cheryan S., Ziegler S.A., Montoya A.K., Jiang L. (2017). Why are some STEM fields more gender balanced than others?. Psychol. Bull..

[16] Cimpian J.R., Kim T.H., McDermott Z.T. (2020). Understanding persistent gender gaps in STEM. Science.

[17] Moss-Racusin C.A., Sanzari C., Caluori N., Rabasco H. (2018). Gender bias produces gender gaps in STEM engagement. Sex Roles.

[18] Delaney J.M., Devereux P.J. (2019). Understanding gender differences in STEM: Evidence from college applications✰. Econ. Educ. Rev..

[19] Zhang Y., Gros T., Mao E. (2021). Gender disparity in students’ choices of information technology majors. Bus. Syst. Res. Int. J. Soc. Adv. Innov. Res. Econ..

[20] Blackburn H. (2017). The status of women in STEM in higher education: A review of the literature 2007–2017. Sci. Technol. Libr..

[21] Sullivan A.A. (2019). Breaking the STEM Stereotype: Reaching Girls in Early Childhood.

[22] Master A., Meltzoff A.N. (2016). Building bridges between psychological science and education: Cultural stereotypes, STEM, and equity. Prospects.

[23] Steele C.M., Aronson J. (1995). Stereotype threat and the intellectual test performance of African Americans. J. Pers. Soc. Psychol..

[24] Su R., Rounds J., Armstrong P.I. (2009). Men and things, women and people: A meta-analysis of sex differences in interests. Psychol. Bull..

[25] Borsotti V. Barriers to gender diversity in software development education: Actionable insights from a Danish case study. Proceedings of the 40th International Conference on Software Engineering: Software Engineering Education and Training.

[26] Botturi L., Bramani C., McCusker S. (2012). Boys are like girls: Insights in the gender digital divide in higher education in Switzerland and Europe. Int. J. Univers. Comput. Sci. Educ..

[27] Ferguson S.J. (2016). Women and Education: Qualifications, Skills and Technology. Women in Canada: A Gender-Based Statistical Report. Stat. Can..

[28] Gargallo-Castel A., Esteban-Salvador L., Pérez-Sanz J. (2010). Impact of gender in adopting and using ICTs in Spain. J. Technol. Manag. Innov..

[29] Berkowsky R., Cotton S. (2015). Igning Off: Predicting Discontinued ICT Usage Among Older Adults in Assisted and Independent Living. Proceedings of the International Conference on Human Aspects of IT for the Aged Population.

[30] Neves B.B., Franz R.L., Munteanu C., Baecker R. (2017). Adoption and Feasibility of a Communication App to Enhance Social Connectedness amongst Frail Institutionalized Oldest Old: An Embedded Case Study. Inf. Commun. Soc..

[31] Vines J., Pritchard G., Wright P., Olivier P., Brittain K. (2015). An Age-old Problem: Examining the Discourses of Ageing in HCI and Strategies for Future Research. ACM Trans. Comput.–Hum. Interact..

[32] Vroman K.G., Arthanat S., Lysack C. (2015). “Who Over 65 is Online?” Older Adults’ Dispositions toward Information Communication Technology. Comput. Hum. Behav..

[33] Friemel T.N. (2014). The digital divide has grown old: Determinants of a digital divide among seniors. New Media Soc..

[34] Hunsaker A., Hargittai E. (2017). A review of Internet use among older adults. New Media Soc..

[35] Laor T., Lissitsa S., Galily Y. (2019). Online digital Radion apps usages in Israel: Consumers, consumption and meaning. Technol. Soc..

[36] Ballano S., Uribe A.C., Munté-Ramos R.À. (2014). Young users and the digital divide: Readers, participants or creators on Internet?. Commun. Soc..

[37] Neves B.B., Waycott J., Malta S. (2018). Old and afraid of new communication technologies? Reconceptualising and contesting the ‘age-based digital divide’. J. Sociol..

[38] Armfield G.G., Holbert R.L. (2003). The relationship between religiosity and internet use. J. Media Relig..

[39] Putnam R.D., Campbell D.E. (2018). American Grace: How Religion Divides and Unites US.

[40] Charlton J.P., Soh P.C.-H., Ang P.H., Chew K.-W. (2013). Religiosity, adolescent internet usage motives and ad nbdiction: An exploratory study. Inf. Commun. Soc..

[41] Campbell H. (2006). Religion and the Internet. Commun. Res. Trends.

[42] Campbell H.A. (2013). Religion and the Internet: A microcosm for studying Internet trends and implications. New Media Soc..

[43] Swatos W.H., Christiano K.J., Swatos W., Olson D. (2000). Secularization theory. Secul. Debate.

[44] Hwang Y., Se-Hoon J. (2009). Revisiting the knowledge gap hypothesis: A meta-analysis of thirty-five years of research. Journal. Mass Commun. Quarterly.

[45] Katz E., Blumler J., Gurevitch M. (1973). Uses and gratifications research. Public Opin. Q..

[46] Galily Y. (2018). Artificial intelligence and sports journalism: Is it a sweeping change?. Technol. Soc..

[47] Galily Y. (2019). Shut up and dribble!? Athletes activism in the age of twittersphere: The case of LeBron James. Technol. Soc..

